# The Korea *Brassica* Genome Project: a Glimpse of the*  Brassica *Genome Based on Comparative Genome Analysis With *Arabidopsis*

**DOI:** 10.1002/cfg.465

**Published:** 2005-04

**Authors:** Tae-Jin Yang, Jung-Sun Kim, Ki-Byung Lim, Soo-Jin Kwon, Jin-A Kim, Mina Jin, Jee Young Park, Myung-Ho Lim, Ho-Il Kim, Seog Hyung Kim, Yong Pyo Lim, Beom-Seok Park

**Affiliations:** 1 National Institute of Agricultural Biotechnology (NIAB) 224 Suinro Gwonseon-gu Gyeonggi-do Suwon 441–707 Korea; 2 Chungnam National University Gungdong 220 Chungnam Daejeon 305–764 Korea

## Abstract

A complete genome sequence provides unlimited information in the sequenced organism as well as in related taxa. According to the guidance of the Multinational Brassica Genome Project (MBGP), the Korea Brassica Genome Project (KBGP) is sequencing chromosome 1 (cytogenetically oriented chromosome #1) of *Brassica rapa*. We have selected 48 seed BACs on chromosome 1 using EST genetic markers and FISH analyses. Among them, 30 BAC clones have been sequenced and 18 are on the way. Comparative genome analyses of the EST sequences and sequenced BAC clones from *Brassica* chromosome 1 revealed their homeologous partner regions on the *Arabidopsis* genome and a syntenic comparative map between *Brassica* chromosome 1 and *Arabidopsis* chromosomes. *In silico* chromosome walking and clone validation have been successfully applied to extending sequence contigs based on the comparative map and BAC end sequences. In addition, we have defined the (peri)centromeric heterochromatin blocks with centromeric tandem repeats, rDNA and centromeric retrotransposons. In-depth sequence analyses of five homeologous BAC clones and an *Arabidopsis* chromosomal region reveal overall co-linearity, with 82% sequence similarity. The data indicate that the *Brassica* genome has undergone triplication and subsequent gene losses after the divergence of *Arabidopsis* and *Brassica*. Based on in-depth comparative genome analyses, we propose a comparative genomics approach for conquering the *Brassica* genome. In 2005 we intend to construct an integrated physical map, including sequence information from 500 BAC clones and integration of fingerprinting data and end sequence data of more than 100 000 BAC clones. The sequences have been submitted to GenBank with accession numbers: 10 204 BAC ends of the KBrH library (CW978640–CW988843); KBrH138P04, AC155338; KBrH117N09, AC155337; KBrH097M21, AC155348; KBrH093K03, AC155347; KBrH081N08, AC155346; KBrH080L24, AC155345; KBrH077A05, AC155343; KBrH020D15, AC155340; KBrH015H17, AC155339; KBrH001H24, AC155335; KBrH080A08, AC155344; KBrH004D11, AC155341; KBrH117M18, AC146875; KBrH052O08, AC155342.


**Comparative and Functional Genomics**
*Comp Funct Genom* 2005; **6**: 138–146.
Published online in Wiley InterScience (www.interscience.wiley.com). **DOI:** 10.1002/cfg.465

**Conference Paper**

### The Korea *Brassica* Genome Project: A glimpse of the *Brassica* genome based on comparative genome analysis with *Arabidopsis*

Tae-Jin Yang[1], Jung-Sun Kim[1], Ki-Byung Lim[1], Soo-Jin Kwon[1], Jin-A Kim[1], Mina Jin[1], Jee Young Park[1], Myung-Ho Lim[1], Ho-Il Kim[1], Seog Hyung Kim[1], Yong Pyo Lim[2] and Beom-Seok Park[1]*

[1] *National Institute of Agricultural Biotechnology (NIAB), 224 Suinro Gwonseon-gu, Suwon, Gyeonggi-do, 441–707, Republic of Korea*
[2] *Chungnam National University, Gungdong 220, Daejeon, Chungnam, 305–764, Republic of Korea*

**Correspondence to:*
*Beom-Seok Park, National Institute of Agricultural Biotechnology (NIAB), 224 Suinro Gwonseon-gu, Suwon, Gyeonggi-do 441-707, Republic of Korea.*
*E-mail: pbeom@rda.go.kr*

### Abstract

**A complete genome sequence provides unlimited information in the sequenced organism as well as in related taxa. According to the guidance of the Multinational Brassica Genome Project (MBGP), the Korea Brassica Genome Project (KBGP) is sequencing chromosome 1 (cytogenetically oriented chromosome #1) of *Brassica rapa*. We have selected 48 seed BACs on chromosome 1 using EST genetic markers and FISH analyses. Among them, 30 BAC clones have been sequenced and 18 are on the way. Comparative genome analyses of the EST sequences and sequenced BAC clones from *Brassica* chromosome 1 revealed their homeologous partner regions on the *Arabidopsis* genome and a syntenic comparative map between *Brassica* chromosome 1 and *Arabidopsis* chromosomes. *In silico* chromosome walking and clone validation have been successfully applied to extending sequence contigs based on the comparative map and BAC end sequences. In addition, we have defined the (peri)centromeric heterochromatin blocks with centromeric tandem repeats, rDNA and centromeric retrotransposons. In-depth sequence analyses of five homeologous BAC clones and an *Arabidopsis* chromosomal region reveal overall co-linearity, with 82% sequence similarity. The data indicate that the *Brassica* genome has undergone triplication and subsequent gene losses after the divergence of *Arabidopsis* and *Brassica*. Based on in-depth comparative genome analyses, we propose a comparative genomics approach for conquering the *Brassica* genome. In 2005 we intend to construct an integrated physical map, including sequence information from 500 BAC clones and integration of fingerprinting data and end sequence data of more than 100 000 BAC clones. The sequences have been submitted to GenBank with accession numbers: 10 204 BAC ends of the KBrH library (CW978640–CW988843); KBrH138P04, AC155338; KBrH117N09, AC155337; KBrH097M21, AC155348; KBrH093K03, AC155347; KBrH081N08, AC155346; KBrH080L24, AC155345; KBrH077A05, AC155343; KBrH020D15, AC155340; KBrH015H17, AC155339; KBrH001H24, AC155335; KBrH080A08, AC155344; KBrH004D11, AC155341; KBrH117M18, AC146875; KBrH052O08, AC155342.** **Copyright © 2005 John Wiley & Sons, Ltd.**

Received: 21 January 2005
Accepted: 2 February 2005

**Keywords:** **KBGP; *Brassica rapa*; genome sequencing; integrated physical mapping; comparative genomics; *in silico*; BAC end sequences**

Copyright © 2005 John Wiley & Sons, Ltd.

### Introduction

The *Arabidopsis* genome has been sequenced completely by an international consortium (the Arabidopsis Genome Initiative, 2000). *Arabidopsis* and *Brassica* diverged 14.5–20.4 million years ago from a common ancestor (Bowers *et al.*, 2003). Comparative genetic mapping has revealed co-linear chromosome segments (Kowalski *et al.*, 1994; Lagercrantz *et al.*, 1996; Paterson *et al.*, 2000, 2001; Schmidt *et al.*, 2001) in the family Brassicaceae and linkage arrangements between *Arabidopsis* and *B. oleracea* (Lukens *et al.*, 2003). The genomes of *Brassica* species have duplicated, perhaps triplicated, counterparts of the corresponding homeologous segments of *Arabidopsis* (O'Neill and Bancroft, 2000; Rana *et al.*, 2004).

*Brassica* is one of the core genera in the family *Brassicaceae*. Six *Brassica* species are cultivated worldwide; three diploids: *B. rapa* (AA, $2n = 20$), *B. nigra* (BB, $2n = 16$) and *B. oleracea* (CC, $2n = 18$), and three amphidiploids (allotetraploids): *B. juncea* (AABB, $2n = 36$), *B. napus* (AACC, $2n = 38$) and *B. carinata* (BBCC, $2n = 34$) (U. 1935). The species *B. rapa* (syn. *campestris*), with 529 Mb per haploid genome equivalent (Johnston *et al.*, 2005), was prioritized for sequencing by a multinational collaboration. The Multinational *Brassica* Genome Project (MBGP) and *Brassica rapa* Genome Sequencing Project (BrGSP) are aiming to completely sequence the genome of *Brassica rapa* inbred line 'Chiifu' (**http://www.brassicagenome.org**; **http://www.brassica-rapa.org**). Korea launched the Korea *Brassica* Genome Project (KBGP) for complete sequencing of the cytogenetic chromosome 1 using BAC-by-BAC shotgun sequencing. In-depth comparative sequence analyses of the sequenced *B. rapa* BAC clones revealed overall co-linearity with a homeologous region of the *Arabidopsis* genome. Comparative sequence analyses suggest that we can use the *Arabidopsis* genome as a backbone for *in silico* clone validation of seed BAC clones and physical mapping as in the report of Love *et al.*, 2004.

Here we propose an efficient clone validation method for selecting chromosome-specific seed BACs using comparative physical mapping and BAC end sequences. In 2005, KBGP aims to sequence 500 BAC clones that correspond to the majority of *Arabidopsis* euchromatin regions. The 500 BACs will be distributed and mapped on *B. rapa* chromosomes through sequence tagged site (STS) or simple sequence repeat (SSR) markers. BAC end sequences of 100 000 BACs (STC) and fingerprinting polymorphism-based BAC contigs (FPC) will be available soon. Hence, the sequence and map information of 500 BACs can be integrated with STCs and FPCs, resulting in an integrated physical map. The integrated physical map will provide a high resolution genome wide comparative map with *Arabidopsis* and will be supplied to MBGP to accelerate the *Brassica* genome sequencing.

### Materials and methods

### DNA sequencing

Shotgun sequencing libraries were constructed in pCUGIblu31 for average insert size of 3 kb (Kim *et al.*, 2004; Yang *et al.*, 2004; Yang *et al.*, 2005). BigDye terminators chemistry v3.0 (ABI) was used for the reactions. The sequences were analysed using ABI3730 automatic DNA sequencers (ABI). Base-calling was performed automatically using phred, and vector sequences were removed by CROSS_MATCH (Ewing and Green 1998; Ewing *et al.*, 1998). High quality, vector-trimmed sequences were thus used for the sequence assembly of each BAC clone, using phrap and consed (Gordon *et al.*, 1998).

### Sequence analysis

Pairwise sequence comparison was conducted using PipMaker (Schwartz *et al.*, 2000) and BLAST2 analysis (**http://www.ncbi.nlm.nih.gov/BLAST/**). MegaBLAST against the *Arabidopsis* chromosome database and BLAST-nr were used as needed (**http://www.ncbi.nlm.nih.gov/BLAST/**). Gene annotation was achieved using several web based gene prediction programs, e.g. FGENE-SH *Arabidopsis* (**http://www.softberry.com/berry.phtml**) and GeneMark *Arabidopsis* (**http://opal.biology.gatech.edu/GeneMark/eukhmm.cgi**).
Repeats were identified using Repeatmasker (**http://ftp.genome.washington.edu/RM/webrepeatmaskerhelp.html**).

Copyright © 2005 John Wiley & Sons, Ltd.

### Fluorescence *in situ* hybridization

Our FISH protocol was adapted from Lim *et al.*, (2001, 2005a) with minor modifications. FISH signals were pseudo-coloured and further improved for optimal brightness and contrast with Adobe Photoshop image processing software.

### Results and discussion

### Overview of *Brassica rapa* genome structure

A genetic map of *Brassica rapa*, using segregating doubled haploid lines of Chiifu and Kenshin, covering 1046 cM with 494 markers on 10 linkage groups, was constructed with 895 DNA markers, AFLP, PCR-RFLP, ESTP, CAPS and SSR (**http://www.brassicagenome.org**). We have constructed another EST-RFLP genetic map of *B. rapa* using 478 tissue-specific cDNA clones consisting of 176 cDNAs from immature flowers, 252 cDNAs from anthers and 50 from dark-grown seedlings of *B. rapa* ssp. *pekinensis* cv. Jangwon. This molecular map covered 3412 cM on 10 linkage groups. Aligning RFLP marker sequences on the counterpart *Arabidopsis* chromosomes shows syntenic co-linearity, resulting in a highly informative comparative genetic map (Kim, 2001). The karyotypes of *B. rapa* chromosomes were studied previously (Fukui *et al.*, 1998; Snowdon *et al.*, 2002; Koo *et al*, 2004). We further characterized chromosomes in detail using fluorescence *in situ* hybridization (FISH) using repetitive DNAs, such as 45S rDNA, 5S rDNA, centromeric repeats (CentBr) and centromere-specific retrotransposons (Lim *et al.*, 2005a). The cytogenetic chromosomes were integrated with genetic maps by painting with chromosome-specific BAC clones identified by unique EST clones from each linkage group (LG1–LG10) (Lim *et al.*, 2005b). The cytogenetic chromosome numbers, our linkage groups (LG1–LG10) and the international standard linkage numbers (R1–R10) (Lombard and Delourme, 2001) will be integrated soon.

We have sequenced four BAC clones that form the counterpart of an *Arabidopsis* chromosomal region (chromosome 5: 3.1–3.2 Mb) containing flowering locus C (FLC). Comparisons of the sequenced *Brassica* BAC clones with the homeologous regions of *Arabidopsis* showed overall co-linearity with 81% sequence similarity. The average sequence similarity between *Brassica* BACs is 82% with exceptionally high similarity (97%) of two clones, 117M18 and 52O08, representing two regions that have recently been duplicated. The co-linear 125 kb *Arabidopsis* sequence was reduced by up to 40% by deletions of DNA segments in *Brassica* BAC clones (Table 1). Among 36 genes in the 125 kb of *Arabidopsis* sequence, only 24, 17, 13, and 13 homologues remained in the common sequence of each BAC clone, 80A08, 4D11, 52O08 and 117M18, respectively. Only four genes remain in all four BAC clones, with 77–96% similarity in amino acid sequences. Newly emerged (or inserted)

**Table 1.** Comparison of four homologous *Brassica* BAC clones and its counterpart *Arabidopsis* sequence

| | **Homologous *Brassica* BAC clones** | | | | |
|---|---|---|---|---|---|
| **Subject** | **80A08** | **4D11** | **52O08** | **117M18** | ***Arabidopsis* chrom. 5** |
| Insert size (bp) | 110 219 | 106 476 | 153 587 | 132 883 | — |
| Common sequence (begin) | 1 | 15 001 | 58 254 | 18 227 | 3 134 987 |
| Common sequence (end) | 110 219 | 89 318 | 115 292 | 70 502 | 3 258 842 |
| Total length of common sequence (bp) | 110 217 | 74 316 | 57 037 | 52 274 | 123 855[b] |
| Aligned nucleotide (bp)[a] | 67 412 | 47 325 | 33 870 | 31 161 | |
| Internal deletion or substitution (bp) | 42 795 | 26 991 | 23 167 | 21 113 | |
| Co-linearity index[b] | 0.9 | 0.6 | 0.5 | 0.4 | 1.0 |
| Alignment index[c] | 0.61 | 0.64 | 0.59 | 0.60 | |
| Homology (*Arabidopsis* vs. Others) (%) | 81.0 | 81.1 | 81.7 | 81.3 | 100.0 |
| Homology (117M18 vs. Others) (%) | 82.3 | 81.8 | 98.1 | 100.0 | 82.3 |

[a] A total of nucleotides that show significant sequence similarity with co-linear *Arabidopsis* sequence.

[b] represents genome expanding or reducing in *Brassica* BAC clones compared to the co-linear *Arabidopsis* sequence (= Total length of common sequence of *Brassica*/Common sequence of *Arabidopsis*.

[c] represents the significantly homeologous region in the common sequence (= Aligned nucleotide/Total length of common sequence.

Copyright © 2005 John Wiley & Sons, Ltd. 

genes including transposons are detected six, three, two and one times in each BAC clone, respectively. The data support previous reports (O'Neill and Bancroft, 2000; Rana *et al*., 2004) and provide in depth information about how triplicated *Brassica* genome sequences are modified after divergence with *Arabidopsis* at around 20 million years ago (Bowers *et al*., 2003).

### Pericentromeric heterochromatin blocks in the *Brassica rapa* genome

The centromeric region of *Brassica* is occupied by 176 bp tandem repeats (Harrison and Heslop-Harrison, 1995). The 176 bp centromeric repeat of *Brassica* (named CentBr) occurred in 30% of our BAC end sequences (10 204 BAC ends of the KBrH library; GenBank accession numbers CW978 640–CW988 843) as tandem arrays, indicating that the CentBr is a major component of the *B. rapa* centromere. The CentBr sequences are subdivided into two classes, named CentBr1 and CentBr2, based on sequence similarity (82–84% between two classes and over 92% between members in each class). CentBr1 and CentBr2 occupy the centromeres of eight and two chromosomes, respectively (Lim *et al*., 2005a).

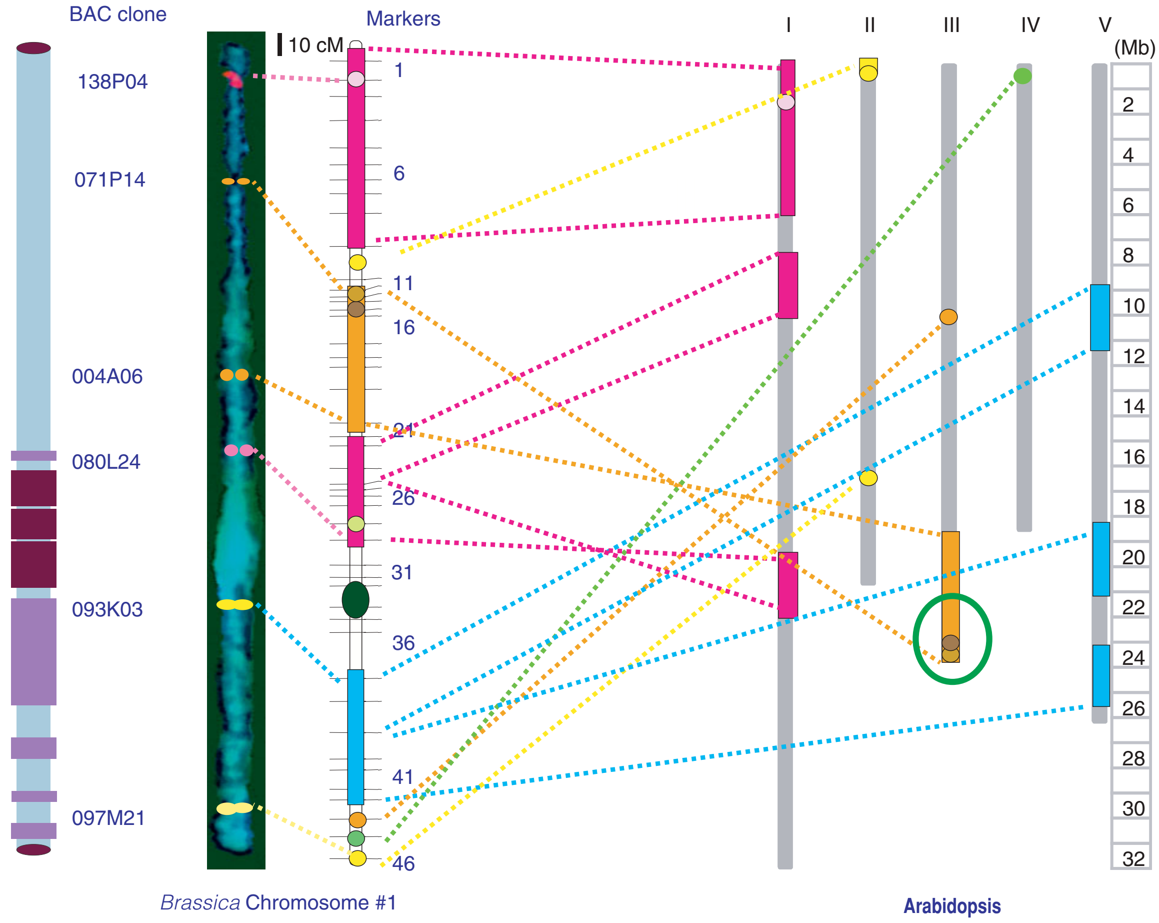

**Figure 1.** Comparative map of *B. rapa* chromosome 1 and *Arabidopsis* chromosomes based on sequence similarity of EST markers and sequenced BAC clones. The far left of the figure represents the features of chromosome 1, BAC clones were selected by filter hybridization using mapped EST markers and their actual chromosomal locations were confirmed by FISH analyses, using metaphase or pachytene phase chromosomes (left). The cartoon at the left of pachytene chromosome represents the features of chromosome 1, showing pericentromeric heterochromatin and heterochromatin (brown and purple boxes, respectively), based on numerous inspections by DAPI staining and FISH analyses using repetitive elements. The linkage groups containing 46 markers and corresponding elements of chromosome 1 are represented and the syntenic regions are represented on *Arabidopsis* chromosomes (right)

Copyright © 2005 John Wiley & Sons, Ltd. 

We have sequenced two centromeric BAC clones, KBrH015B20 (102 kb) and KBrH001P13 (17 kb), containing centromeric tandem repeats for increased understanding of major elements in the (peri)centromeric region of the *Brassica* genome. Careful sequence analysis revealed several families of centromere-specific retrotransposons of *Brassica* (CRB). Among these, two long terminal

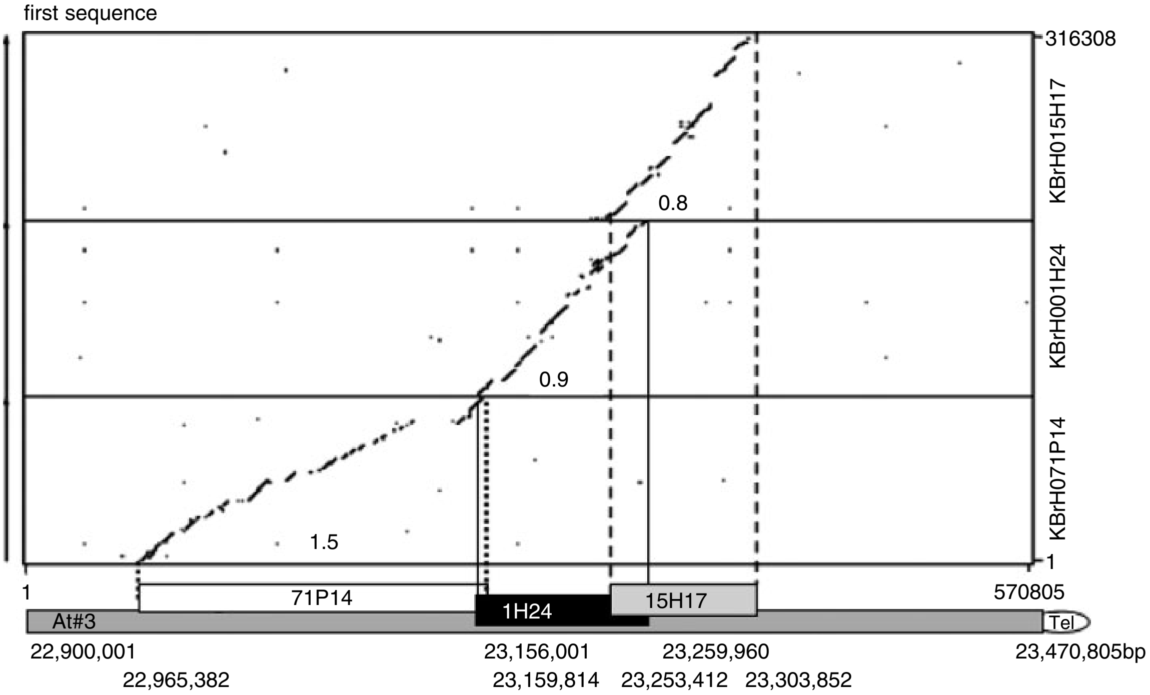

**Figure 2.** Dot-plot analysis of three contiguous sequenced BAC clones from *B. rapa* chromosome 1 and the counterpart region of *Arabidopsis* (chromosome 3, 22.9–23.3 Mb). The region is marked as a green circle in Figure 1. The beginning and ending nucleotide of the counterpart *Arabidopsis* sequence for the three *Brassica* BAC clones are represented as numericals under the figure. The co-linear index (= The length of co-linear *Arabidopsis* sequence/Co-linear *Brassica* BAC sequence which is in accordance with the slope) of each BAC clone is represented in the dot plot

**Table 2.** *Brassica* BAC clones sequenced and the counterpart *Arabidopsis* sequence

| ***Brassica* BAC Clone** | | **Co-linear Index** | **Counterpart sequence in *Arabidopsis*** | | | |
|---|---|---|---|---|---|---|
| **Name** | **Length** | ***Arabidopsis*/*Brassica*** | **Length** | **Begin** | **End** | **Chrom. No** |
| 01H24 | 118 144 | 0.9 | 103 420 | 23 156 540 | 23 259 960 | 3 |
| 15H17 | 110 885 | 0.8 | 85 302 | 23 224 319 | 23 309 621 | 3 |
| 20D15 | 143 633 | 0.8 | 119 117 | 8 047 497 | 8 166 614 | 1 |
| 77A05 | 113 253 | 1.1 | 125 641 | 19 236 565 | 19 362 206 | 5 |
| 80L24 | 115 119 | 4.0 | 459 225 | 17 701 122 | 18 160 347 | 1 |
| 97M21 | 131 063 | 2.4 | 314 999 | 10 677 001 | 10 992 000 | 3 |
| 117N09 | 125 390 | 1.7 | 218 929 | 17 478 001 | 17 696 930 | 2 |
| 138P04 | 137 697 | 1.3 | 173 013 | 2 128 977 | 2 301 990 | 1 |
| 4D11 | 106 476 | 1.7 | 184 272 | 3 092 144 | 3 276 416 | 5 |
| 80A08 | 110 038 | 1.1 | 121 376 | 3 137 466 | 3 258 842 | 5 |
| Average insert size (bp) | 121 170 | 1.6 | 190 529 | | | |
| STD | ±12 682 | 1.0 | 116 235 | | | |

Copyright © 2005 John Wiley & Sons, Ltd. 

repeat (LTR) retrotransposons, a Ty3-gypsy-like one (PCRB; 9135 bp with 2047bp LTR) and a Ty1-copia-like one (CRB; 6010 bp with 597 bp LTR) predominantly occupied each BAC sequence. FISH analyses revealed that the CRB is a major component of the centromere of all chromosomes and the PCRB is a major component of the large pericentromeric heterochromatin regions of three chromosomes. Based on the BAC end sequence information and FISH analyses, we assume these four (peri)centromeric repeats occupy just over 40% of the *Brassica* genome. Since heterochromatin blocks are hard to sequence, we will focus on sequencing of euchromatin regions, which probably constitute less than 60% of the *Brassica rapa* genome.

### Progress in sequencing chromosome 1 in Korea

The Korea *Brassica* Genome Project (KBGP) is aiming to complete the sequencing of cytogenetic chromosome 1 using three BAC libraries, KBrH (*Hin*dIII), KBrB (*Bam*HI), and KBrS (*Sau*3AI), of *B. rapa* ssp. *pekinensis* inbred line 'Chiifu'. Physical mapping is on-going by fingerprinting of the KBrH and KBrB libraries (**http://www.brassicagenome.org; http://www.brassica-rapa.org**). Anchoring the fingerprint polymorphism contigs (FPC) on the chromosome remains an obstacle to overcome for physical mapping and clone validation. We have selected 48 seed BACs on chromosome 1 through screening with EST markers and confirmation by FISH analyses. Among them, 30 BAC clones were sequenced and they show co-linearity with the counterpart homeologous region of *Arabidopsis*, with about 82% sequence similarity (Table 1).

The comparative analyses of the EST sequences mapped on chromosome 1 with their homeologous partner regions of *Arabidopsis* revealed counterparts in the *Arabidopsis* genome (Figure 1). The sequenced BAC clones show overall co-linearity with a counterpart *Arabidopsis* chromosomal region which was expected, based on the

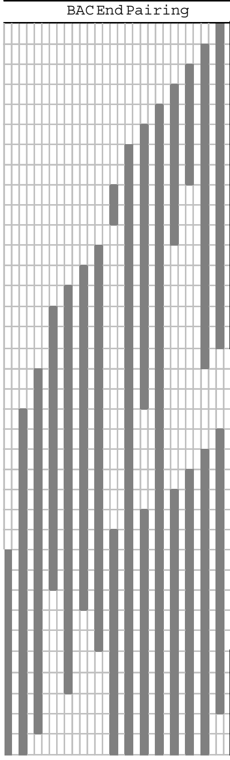

| BAC Ends | Direction | Length | Matching | | | Span (bp) | Arabidopsis Match Region | | | GenBank No. | E Value |
|---|---|---|---|---|---|---|---|---|---|---|---|
| **KBrB092L06R** | **+** | **792** | **121** | - | **764** | **207,171** | **441,816** | - | **442,459** | NC_003070.5 | **1.00E-119** |
| KBrB078D07F | + | 660 | 228 | - | 495 | 200,497 | 449,221 | - | 449,488 | NC_003070.5 | 3.00E-78 |
| KBrB069J20F | + | 852 | 229 | - | 500 | 55,796 | 449,221 | - | 449,492 | NC_003070.5 | 1.00E-99 |
| KBrB072O20R | + | 561 | 242 | - | 513 | 162,673 | 449,221 | - | 449,492 | NC_003070.5 | 4.00E-95 |
| KBrB064C19F | + | 367 | 188 | - | 277 | 438,409 | 456,834 | - | 456,923 | NC_003070.5 | 3.00E-27 |
| KBrH014E03F | + | 767 | 677 | - | 754 | 175,318 | 486,300 | - | 486,377 | NC_003070.5 | 2.00E-08 |
| KBrB006N24R | + | 752 | 227 | - | 472 | 409,867 | 489,141 | - | 489,386 | NC_003070.5 | 3.00E-41 |
| KBrB069J20R | - | 342 | 1 | - | 126 | | 505,017 | - | 504,892 | NC_003070.5 | 2.00E-46 |
| KBrS006E09F | + | 792 | 1 | - | 87 | 41,204 | 567,509 | - | 567,597 | NC_003070.5 | 4.00E-19 |
| KBrS006E09R | - | 686 | 197 | - | 514 | | 608,713 | - | 608,396 | NC_003070.5 | 1.00E-120 |
| KBrB072O20F | - | 346 | 1 | - | 222 | | 611,894 | - | 611,674 | NC_003070.5 | 7.00E-25 |
| KBrB092L16R | + | 665 | 159 | - | 372 | 120,321 | 612,015 | - | 612,234 | NC_003070.5 | 4.00E-31 |
| KBrB001E05F | + | 698 | 8 | - | 687 | 107,536 | 618,404 | - | 619,098 | NC_003070.5 | 1.00E-147 |
| **KBrB030F10R** | **+** | **736** | **73** | - | **123** | **129,058** | **624,089** | - | **624,139** | NC_003070.5 | **0.0001** |
| KBrH008B01F | + | 794 | 150 | - | 497 | 85,749 | 638,255 | - | 638,602 | NC_003070.5 | 5.00E-83 |
| **KBrB092L06F** | **-** | **845** | **783** | - | **845** | | **648,987** | - | **648,925** | NC_003070.5 | **2.00E-15** |
| KBrB078D07R | - | 824 | 277 | - | 642 | | 649,718 | - | 649,360 | NC_003070.5 | 3.00E-72 |
| KBrB049O03F | + | 802 | 161 | - | 201 | 105,990 | 651,701 | - | 651,741 | NC_003070.5 | 6.00E-12 |
| KBrH014E03R | - | 849 | 35 | - | 281 | | 661,618 | - | 661,372 | NC_003070.5 | 3.00E-44 |
| KBrH006J03R | + | 711 | 145 | - | 221 | 163,565 | 666,344 | - | 666,420 | NC_003070.5 | 5.00E-12 |
| KBrS011G09F | + | 796 | 450 | - | 605 | 87,764 | 667,143 | - | 667,298 | NC_003070.5 | 2.00E-30 |
| KBrS016E11R | + | 699 | 167 | - | 275 | 120,558 | 681,105 | - | 681,213 | NC_003070.5 | 6.00E-24 |
| KBrS005M08R | + | 464 | 2 | - | 206 | 128,380 | 681,713 | - | 681,917 | NC_003070.5 | 1.00E-33 |
| KBrB021O04F | + | 838 | 643 | - | 822 | 266,085 | 685,555 | - | 685,734 | NC_003070.5 | 1.00E-37 |
| KBrB069F04F | + | 850 | 644 | - | 823 | 266,085 | 685,555 | - | 685,734 | NC_003070.5 | 1.00E-37 |
| KBrB042P04R | + | 532 | 1 | - | 189 | 283,170 | 709,677 | - | 709,862 | NC_003070.5 | 1.00E-58 |
| KBrS005C04R | + | 719 | 1 | - | 719 | 306,547 | 716,206 | - | 716,924 | NC_003070.5 | 0 |
| KBrH008B01R | - | 908 | 657 | - | 905 | | 724,004 | - | 723,756 | NC_003070.5 | 2.00E-64 |
| KBrB001E05R | - | 896 | 435 | - | 828 | | 725,940 | - | 725,548 | NC_003070.5 | 1.00E-93 |
| KBrB056O02F | + | 821 | 126 | - | 223 | 86,208 | 728,279 | - | 728,373 | NC_003070.5 | 4.00E-13 |
| KBrB092L16F | - | 942 | 6 | - | 540 | | 732,336 | - | 731,793 | NC_003070.5 | 1.00E-115 |
| **KBrB089B13R** | **+** | **839** | **1** | - | **242** | **341,507** | **732,356** | - | **732,597** | NC_003070.5 | **3.00E-60** |
| **KBrB030F10F** | **-** | **858** | **769** | - | **844** | | **753,147** | - | **753,072** | NC_003070.5 | **7.00E-21** |
| KBrS011G09R | - | 721 | 269 | - | 712 | | 754,907 | - | 754,465 | NC_003070.5 | 1.00E-140 |
| KBrB049O03R | - | 683 | 54 | - | 196 | | 757,691 | - | 757,549 | NC_003070.5 | 3.00E-44 |

**Figure 3.** *In silico* allocation of *Brassica* BAC clones on *Arabidopsis* chromosomes. The beginning part of *Arabidopsis* chromosome 1 is represented. BAC clones are aligned on *Arabidopsis* chromosomes based on significant and directional matches of both ends within a 30–500 kb interval. The forward and reverse ends are marked as grey bars (left). An example of the minimum tiling path of the three BAC clones are boxed

Copyright © 2005 John Wiley & Sons, Ltd. 

comparative map (Figure 2). Based on the comparative physical map and micro-co-linearity between the *Brassica* and *Arabidopsis* sequences, we have proposed an efficient and novel clone validation method for sequencing in advance of the complete physical map. The *Brassica* BAC clones were allocated to *Arabidopsis* chromosomes by *in silico* allocation based on unique, significant (<1E-6), and directional matches: one BAC end is forward and the other end is the reverse, with a complement match within a 30–500 kb interval. BAC-FISH and STS mapping using BAC end sequences on the counterpart *Arabidopsis* chromosomal region showed the real locations of the BAC clones on the chromosomes. At least one in three BAC clones is mapped onto the expected region of chromosome 1 due to the triplicated nature of the *Brassica* genome. All the sequenced BAC clones provide a further starting point for selection of seed BAC clones for extending the sequence.

### Integrated physical mapping

Successful clone validation based on *in silico* allocation to counterparts of chromosome 1 suggests a novel strategy for integrated physical mapping, using comparative mapping of BAC ends onto *Arabidopsis* chromosomes. The integrated physical mapping strategy encompasses *in silico* allocation of *B. rapa* BAC clones to the counterpart locations of *Arabidopsis* chromosomes, based on significant BLAST matches. A *Brassica* BAC clone (average size 120 kb) covers an average of 190 kb *Arabidopsis* sequence based on a co-linearity index of 1.6 (= co-linear *Arabidopsis* sequence/*Brassica* BAC nucleotide) (Table 2). We have analysed 91 000 BAC end sequences (Table 3). Among them, a total of 45 232 BAC end sequences (50%) show significant sequence similarity with unique *Arabidopsis* sequences, and a total of 4317 BAC clones (9.5%) are allocated on *Arabidopsis* chromosomes by significant matching with both ends within 30–500 kb interval (Table 3). These 4317 clones span 93 Mb of *Arabidopsis* euchromatin regions, representing 78.2% of the total *Arabidopsis* genome. A total of 26 Mb remain as unconvered gaps: among these 9.4 Mb (3.1 Mb, 1.8 Mb, 2.4 Mb, and 1.0 Mb from *Arabidopsis* chromosomes 1, 2, 3, 4, and 5, respectively) might be from euchromatin gaps at 116 sites ranged from over 20–585 498 bp, except for the 16.6 Mb of pericentromeric heterochromatin gaps. A single *Brassica* BAC clone spans an average of 147 kb (±74 kb) *Arabidopsis* sequence (Figure 3). A total of 500

**Table 3.** *Brassica* rapa BAC end sequence and the results of blast analyses against *Arabidopsis* chromosomes

| | Unique hits (<1E-6) | | | BAC clones with both ends | | |
|---|---|---|---|---|---|---|
| Library | Query No. | Hit No. | % | Pairhit No. | Clone No. | %[a] |
| KBrH (*Hin*dIII) | 10 204 | 4195 | 41.1 | 1162 | 581 | 11.4 |
| KBrB (*Bam*HI) | 72 343 | 36 833 | 50.9 | 6908 | 3454 | 9.5 |
| KBrS (*Sau*3AI) | 8632 | 4204 | 48.7 | 564 | 282 | 6.5 |
| Total | 91 179 | 45 232 | 49.6 | 8634 | 4317 | 9.5 |

[a] 100* (Pairhit No./Query No.). Clone numbers represent the numbers of BAC clones allocated on *Arabidopsis* chromosome by directional hitting with both ends within 30–500 kb interval.

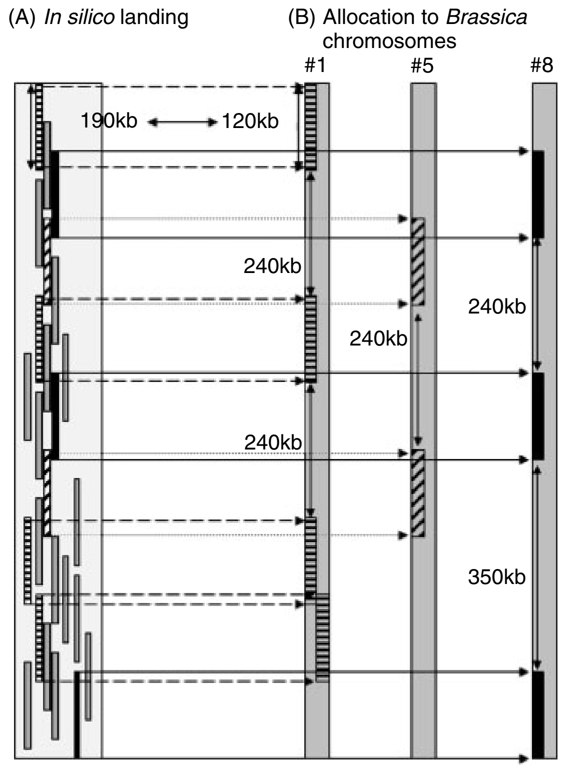

**Figure 4.** Schematic representation of the *in silico* landing on *Arabidopsis* chromosome and estimated real position on *Brassica* chromosomes. Minimum tiled BACs on *in silico* comparative allocation will be scattered onto three *Brassica* chromosomes. If the minimum tiled BACs are sequenced and mapped, fewer than 240 kb physical gaps will remain between BACs in each chromosome

Copyright © 2005 John Wiley & Sons, Ltd. 

BACs with an average 120 kb of insert will cover around 80 Mb of the euchromatin regions of the *Arabidopsis* genome (almost all of the euchromatin). The 500 BACs will be scattered into the triplicated regions on *Brassica* chromosomes (e.g. Figure 4). The actual chromosomal location of a sequenced BAC can be mapped on the genetic map through SSR or STS-PCR using its sequence information. Recently, we have selected the minimum tiled 629 *Brassica* BAC clones spanning 86 Mb of *Arabidopsis* from the *in silico* allocation (data is available at our website: **www.brassica-rapa.org**). Each BAC clone will be mapped on the *Brassica* chromosomes by STS mapping and FISH analyses. About 75 Mb from gene rich euchromatin regions of *Brassica* will be obtained from sequencing of the 629 BACs (average insert 120 kb) that may be distributed into 10 *B. rapa* chromosomes (average 60 BACs for each chromosome) with an average 240 kb gap (Figure 4). All the sequenced BAC clones will be provided to MBGP and used as a starting point for the selection of seed BAC clones extending to the flanking sides with minimum overlap based on sequence tagged connectors (STC). The results will provide in depth information about the comparative genomics between *Brassica* and *Arabidopsis*.

Complete sequencing of *Brassica rapa* will give great opportunities to increase our understanding of the evolution of the polyploidized genome and of agricultural aspects, especially for breeding and molecular farming, through finding novel or useful genes, not only in *B. rapa* but also in other important crops in the genus *Brassica*.

### Acknowledgements

This work was supported by BioGreen 21 Program, RDA, and NIAB, Korea. We are grateful to Macrogen (**http://www.macrogen.com/english/index.html**) for the sequencing.

### References

*Arabidopsis* Genome Initiative. 2000. Analysis of the genome sequence of the flowering plant *Arabidopsis thaliana*. *Nature* **408**: 796–815.

Bowers JE, Chapman AB, Rong Jm, Paterson AH. 2003. Unraveling angiosperm genome evolution by phylogenetic analysis of chromosomal duplication events. *Nature* **422**: 433–438.

Ewing B, Green P. 1998. Base-calling of automated sequencer traces using Phred II. Error probabilities. *Genome Res* **8**: 186–194.

Ewing B, Hillier L, Wendl MC, Green P. 1998. Base-calling of automated sequencer traces using PHRED. I: Accuracy assessment. *Genome Res* **8**: 175–185.

Fukui K, Nakayama S, Ohmido N, Yoshiaki H, Yamabe M. 1998. Quantitative karyotyping of three diploid *Brassica* species by imaging methods and localization of 45S rDNA loci on the identified chromosomes. *Theor Appl Genet* **96**: 325–330.

Gordon D, Abajian C, Green P. 1998. Consed: a graphical tool for sequence finishing. *Genome Res* **8**: 195–202.

Harrison GE, Heslop-Harrison JS. 1995. Centromeric repetitive DNA sequences in the genus *Brassica*. *Theor Appl Genet* **90**: 157–165.

Johnston JS, Pepper AE, Hall AE, *et al*. 2005. Evolution of genome size in Brassicaceae. *Ann Bot* **95**: 229–235.

Kim JS. 2001. Composite of a linkage map of *Brassica rapa* (ssp. *pekinensis*) using clones and comparative study to *Arabidopsis thaliana*. PhD Dissertation, Sungkyunkwan University, Republic of Korea.

Kim HR, Yang TJ, Kudna DA, Wing RA. 2004. Construction and application of genomic DNA libraries. In *Handbook of Plant Biotechnology*, Christou P, Klee H (eds). Wiley: Chichester; 1, 71–80–.

Koo DH, Plaha P, Lim YP, Hur Y, Bang JW. 2004. A high-resolution karyotype of *Brassica rapa* ssp. *pekinensis* revealed by pachytene analysis and multicolour fluorescence *in situ* hybridization. *Theor Appl Genet* **109**: 1346–1352.

Kowalski SD, Lan TH, Feldmann KA, Paterson AH. 1994. Comparative mapping of *Arabidopsis thaliana* and *Brassica olereracea* chromosomes reveals islands of conserved organization. *Genetics* **138**: 499–510.

Lagercrantz U, Putterill J, Coupland G, Lydiate D. 1996. Comparative mapping in *Arabidopis* and *Brassica*, fine scale genome colinearity and congruence of genes controlling flowering time. *Plant J* **9**: 13–20.

Lim KB, Wennekes J, de Jong JH, Jacobsen E, van Tuyl JM. 2001. Karyotype analysis of *Lilium longiflorum* and *Lilium rubellum* by chromosome banding and fluorescence *in situ* hybridization. *Genome* **44**: 911–918.

Lim KB, de Jong H, Yang TJ, Park JY, Jin YM, Park BS. 2005a. Characterisation of rDNAs and tandem repeats in heterochromatin of *Brassica rapa*. *Mol Cells* (in press).

Lim KB, Kim JS, de Jong H, *et al*. 2005b. Development of chromosome-specific markers and integration of genetic map to chromosome map in *Brassica campestris*. XIII Plant and Animal Genome Conference, San Diego, USA 409.

Lombard V, Delourme R. 2001. A consensus linkage map for rapeseed (*Brassica napus* L.): construction and integration of three individual maps from DH populations. *Theor Appl Genet* **103**: 491–507.

Love CG, Batley J, Lim G, *et al*. 2004. New computational tools for *Brassica* genome research. *Comp Funct Genom* **5**: 276–280.

Lukens L, Zou F, Lydiate D, Parkin I, Osborn T. 2003. Comparison of a *Brassica oleracea* genetic map with the genome of *Arabidopsis thaliana*. *Genetics* **164**: 359–372.

O'Neill CM, Bancroft I. 2000. Comparative physical mapping of the segments of the genome of *Brassica oleracea* var. *alboglabra* that are homologous to sequenced regions of chromosomes 4 and 5 of *Arabidopsis thaliana*. *Plant J* **23**: 233–243.

Paterson AH, Bowers JE, Burow MD, *et al*. 2000. Comparative genomics of plant chromosomes. *Plant Cell* **12**: 1523–1539.

Copyright © 2005 John Wiley & Sons, Ltd. 

Paterson AH, Lan TH, Amasino R, Osborn TC, Quiros C. 2001. *Brassica* genomics: a complement to, and early beneficiary of, the *Arabidopsis* sequence. *Genome Biol* **2**: REVIEWS1011.

Rana D, van den Boogaart T, O'Neill CM, *et al.* 2004. Conservation of the microstructure of genome segments in *Brassica napus* and its diploid relatives. *Plant J* **40**: 725–733.

Schmidt R, Acarkan A, Boivin K. 2001. Comparative structural genomics in the Brassicaceae family. *Plant Phys Biochem* **39**: 253–262.

Schwartz S, Zhang Z, Frazer KA, *et al.* 2000. PipMaker — a web server for aligning two genomic DNA sequences. *Genome Res* **10**: 577–586.

Snowdon RJ, Friedrich T, Friedt T, Köhler W. 2002. Identifying the chromosomes of the A- and C-genome diploid *Brassica* species *B. rapa* (syn. *campestris*) and *B. oleracea* in their amphidiploid *B. napus*. *Theor Appl Genet* **104**: 533–538.

UN. 1935. Genome analysis in *Brassica* with special reference to the experimental formation of *B. napus* and peculiar mode of fertilization. *Japan J Bot* **7**: 389–452.

Yang TJ, Lee S, Yu Y, *et al.* 2005. In depth sequence analysis of 326kb centromeric region of tomato chromosome12. *Chromosoma* (in press).

Yang TJ, Yu Y, Frisch D, *et al.* 2004. Construction of various copy number plasmid vectors and their utility for genome sequencing. *Genom Informat* **2**: 153–158.

Copyright © 2005 John Wiley & Sons, Ltd. 
